# Two Older Hematological Malignancy Patients for Whom Nutrition Rehabilitation Was Effective Against Global Leadership Initiative on Malnutrition-Defined Malnutrition and Sarcopenia

**DOI:** 10.7759/cureus.43069

**Published:** 2023-08-07

**Authors:** Takuya Matsunaga, Tatsunori Furuya, Tomoko Deto, Hajime Masuda, Katsuya Nakanishi

**Affiliations:** 1 Department of Hematology, Japan Community Health Care Organization (JCHO) Sapporo Hokushin Hospital, Sapporo, JPN; 2 Department of Rehabilitation, Japan Community Health Care Organization (JCHO) Sapporo Hokushin Hospital, Sapporo, JPN; 3 Department of Nutrition Management, Japan Community Health Care Organization (JCHO) Sapporo Hokushin Hospital, Sapporo, JPN; 4 Department of Diabetes, Japan Community Health Care Organization (JCHO) Sapporo Hokushin Hospital, Sapporo, JPN; 5 Department of Pathology, Japan Community Health Care Organization (JCHO) Sapporo Hokushin Hospital, Sapporo, JPN

**Keywords:** nutrition rehabilitation, hematological malignancy, body mass index, appendicular skeletal muscle mass, malnutrition, global leadership initiative on malnutrition

## Abstract

The long-term survival rate of hematological malignancy patients with Global Leadership Initiative on Malnutrition (GLIM)-defined malnutrition and sarcopenia is poor, but nutritional rehabilitation effects in such patients are unknown. Here, two cases of older hematological malignancy patients in whom nutritional rehabilitation was effective against GLIM-defined malnutrition and sarcopenia are reported. By undergoing nutritional rehabilitation, the myeloma patient increased her six-meter walking speed and her maintained body mass index (BMI), appendicular skeletal muscle mass (ASM), and hand grip strength, whereas the Hodgkin lymphoma patient regained his hand grip strength and maintained his BMI, ASM, and six-meter walking speed.

## Introduction

Global Leadership Initiative on Malnutrition (GLIM)-defined malnutrition [1] and sarcopenia [2] are adverse prognostic factors for patients with hematological malignancies. GLIM-defined malnutrition criteria (GLIM criteria) recommend two steps in the nutritional assessment, which include screening for malnutrition risk using a validated tool and fulfilling both phenotypic and etiologic criteria risk. The malnutrition risk is screened using a validated screening tool such as the Malnutrition Universal Screening Tool (MUST) [3]. Phenotypic malnutrition criteria include unintentional body weight loss, low body mass index (BMI), and low appendicular skeletal muscle mass (ASM) defined using Asia-specific cutoff values (BMI <20.0 kg/m^2^; ASM <7.0 kg/m^2^ in men and <5.7 kg/m^2^ in women, by bioelectrical impedance analysis (BIA)). The etiological criteria for malnutrition have been divided into “decreased food intake/assimilation” and “disease burden/inflammation.” GLIM criteria did not establish any criteria for “disease burden/inflammation.” Multiple myeloma and Hodgkin lymphoma are chronic diseases involving inflammation. Malnutrition severity is confirmed using the following BMI cutoff values: moderate malnutrition, BMI <22.0 kg/m^2^, and severe malnutrition, BMI <20.0 kg/m^2^. GLIM-defined malnutrition has been associated with a higher mortality risk, independent of age and duration of diagnosis, in hospitalized patients with hematologic malignancies, such as lymphoma, leukemia, and myeloma [4].

The Asian Working Group for Sarcopenia 2019 consensus diagnostic criteria (AWGS 2019 criteria) [2] defined sarcopenia as “age-related loss of muscle mass, plus low muscle strength, and/or low physical performance,” without reference to comorbidity. Diagnostic component cut-offs were as follows: low ASM <7.0 kg/m^2^ in men and <5.7 kg/m^2^ in women by BIA. Low muscle strength was defined as hand grip strength <28 kg for men and <18 kg for women. Low physical performance was defined as a six-meter walking speed <1.0 m/second. Sarcopenia was diagnosed when the hand grip strength or six-meter walking speed and ASM met the above criteria. The five-year progression-free survival and overall survival rates are significantly lower in classical Hodgkin lymphoma patients than in those without sarcopenia [5]. Zakaria et al. [6] reported that sarcopenia allows the identification of myeloma patients with spinal metastasis who are at risk of shorter survival.

We previously discussed the usefulness of nutritional rehabilitation for sarcopenia in a patient with diffuse large B-cell lymphoma [7]. The patient regained hand grip strength and maintained ASM. However, the effects of nutritional rehabilitation on GLIM-defined malnutrition in hematological malignancies have not yet been reported. Here, we report the cases of two older patients with hematological malignancies for whom nutrition rehabilitation was effective against GLIM-defined malnutrition and sarcopenia.

## Case presentation

Case one

**Figure 1:**
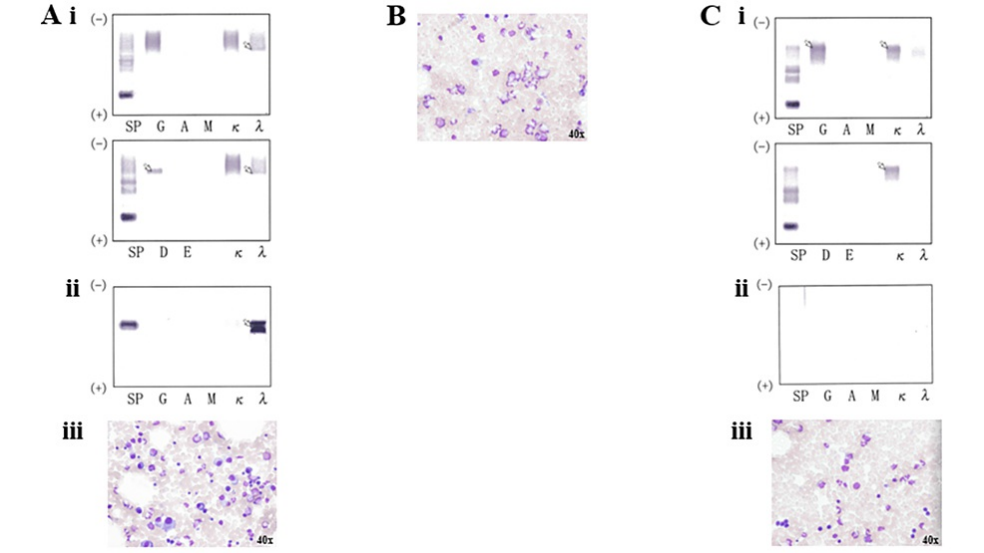
Serum and urine protein electrophoresis and bone marrow aspirate smear findings in Case one. (A) Electrophoresis and bone marrow aspirate smear findings on March 7, 2022. (i) Serum finding, (ii) urine finding, and (iii) bone marrow aspirate smear finding. (B) Bone marrow aspirate smear finding on June 7, 2022. (C) Electrophoresis and bone marrow aspirate smear findings on July 18, 2022. (i) Serum finding, (ii) urine finding, and (iii) bone marrow aspirate smear finding. Arrows indicate M proteins. MayGrundwald-Giemsa staining was performed on the bone marrow aspirate smears.

An 88-year-old woman was referred to our hospital from the Department of Hematology of another hospital in February 2021 for a follow-up of monoclonal gammopathy of undetermined significance. At presentation, she was afebrile, and physical examination revealed no skin changes, lymphadenopathy, or hepatosplenomegaly. The patient was followed up in outpatient visits every three months thereafter. She also had diabetes mellitus, which was treated with oral medications at a clinic near her residence. On March 7, 2022, a complete blood test revealed anemia (hemoglobin (Hb) = 9.2 g/dL; normal range = 13.6-18.3 g/dL). Serum and urine protein electrophoresis showed M protein, immunoglobulin (Ig) D-λ M proteinemia, and positive urinary Bence-Jones protein (Figure 1, Panels Ai, Aii).

**Figure 2:**
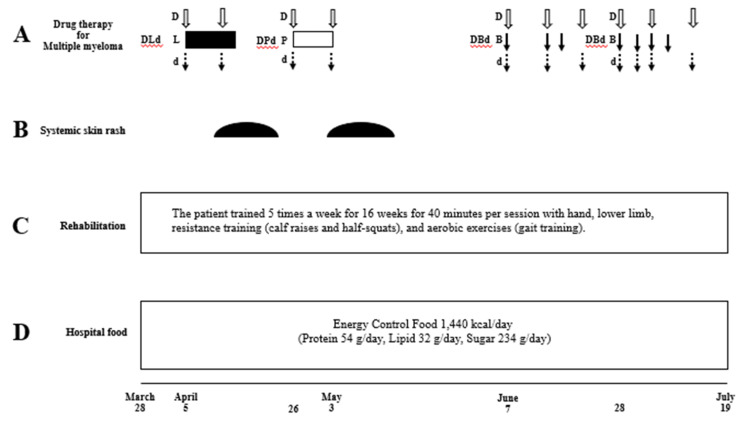
Drug therapy for multiple myeloma, systemic skin rash, and rehabilitation and nutritional therapy in Case one. (A) Drug therapy for multiple myeloma. DLd, daratumumab (D), lenalidomide (L), dexamethazone (d); DPd, daratumumab (D), pomalidomide (P), dexamethazone (d); DBd, daratumumab (D), bortezomib (B), dexamethazone (d). (B) Systemic skin rash occurring as a side effect of drug therapy for multiple myeloma. (C) Rehabilitation protocol. (D) Nutritional therapy using hospital food.

Bone marrow aspirate smear showed plasma cells, accounting for 12.2% of nucleated cells (Figure 1, Panel Aiii). Karyotypic analysis of the bone marrow cells revealed 46, XY. The patient was hospitalized on March 28, 2022 (Figure 2).

Her height, body weight, and ideal body weight were 149.5 cm, 36.3 kg, and 49.2 kg, respectively. An increase in serum HbA1c (8.8%; normal range = 4.6-6.2%), C-reactive protein (CRP = 0.47 mg/dL; normal range = 0.00-0.30 mg/dL), creatinine (Cr = 0.89 mg/dL; normal range = 0.46-0.82 mg/dL), and β2-microglobulin (β-MG = 4.6 mg/L; normal range = 0.9-2.0 mg/L) were observed. However, the estimated glomerular filtration rate (eGFR = 44.9 mL/minute/L; normal range ≥89.0 mL/minute/L) was decreased. Serum albumin (Alb = 4.3 g/dL; normal range = 3.8-5.2 g/dL) and lactate dehydrogenase (LDH = 170 U/mL; normal range = 120-245 U/mL) were within normal limits. Positron emission tomography-computed tomography (PET-CT) showed no osteolytic lesions. The patient had Stage II disease according to the Revised International Staging System for multiple myeloma [8]. We investigated the presence of sarcopenia using the AWGS 2019 criteria. The ASM, hand grip strength, and six-meter walking speed were 5.0 kg/m^2^, 15.4 kg, and 1.31 m/second, respectively. Her ASM and hand grip strength were reduced, and the patient was diagnosed with sarcopenia. Next, we studied the presence of malnutrition using the GLIM criteria. The malnutrition risk is screened using the MUST [3]. The MUST comprises a weight-loss score, BMI score, and acute illness score (total score range = 0-6). A score of 0, 1, or ≥2 indicates no, moderate, or high malnutrition risk, respectively. A MUST score ≥1 was regarded as a malnutrition risk factor in this report. The patient’s MUST scores were 4. There had been no change in her body weight since she first visited our institution. Her BMI was 16.2 kg/m^2^; therefore, she was diagnosed with severe malnutrition.

**Figure 3:**
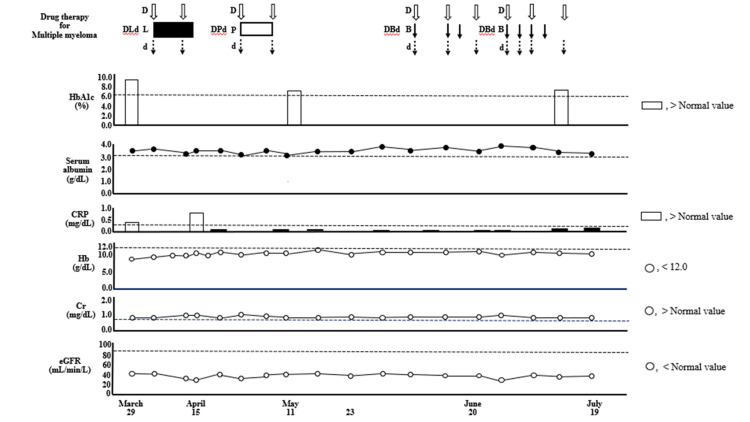
Data of HbA1c, serum Alb, CRP, Hb, Cr, and eGFR in Case one. Alb: albumin; Cr: creatinine; CRP: C-reactive protein; eGFR: estimated glomerular filtration rate; Hb: hemoglobin

**Figure 4:**
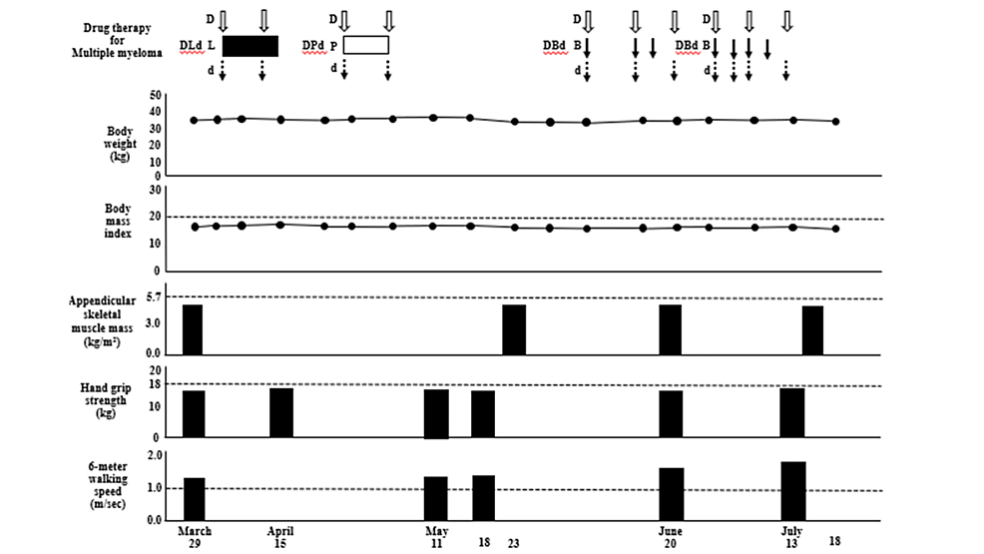
Data of body weight, body mass index, and the Asian Working Group for Sarcopenia 2019 consensus diagnostic criteria in Case one. The criteria for low appendicular skeletal muscle mass is <5.7 kg/m^2^ in women by bioelectrical impedance analysis; the criteria for low muscle strength is defined as hand grip strength <18 kg for women; the criteria for low physical performance is six-meter walking speed <1.0 m/second.

As the patient was diagnosed with severe malnutrition and sarcopenia, a rehabilitation program combining exercise and nutritional intervention was initiated, and chemotherapy was implemented (Figure 2). Diabetes mellitus was treated with a combination of oral medications, fast-acting insulin, and long-acting dissolved insulin analogs. Additionally, to treat multiple myeloma, immunomodulatory drugs and bortezomib, as well as dexamethasone, were administered at a reduced dose because of her advanced age to treat multiple myeloma. After starting DLd therapy [9] (daratumumab (1,800 mg/body; days one and eight), lenalidomide (10 mg/body; days one to ten), and dexamethasone (4 mg/body; days one and eight)) on April 5, 2022, systemic skin rash occurred on April 11, 2022 (Figures 2-4).

Therefore, DLd therapy was discontinued on day 11. Although the serum CRP level improved after the DLd therapy (Figure 3), the patient’s body weight remained unchanged. We administered nutritional therapy (30 kcal/kg ideal body weight/day of energy, 1.1 g/kg ideal body weight/day of protein) using hospital food. The patient consumed energy control food (1,440 kcal/day and 54 g protein/day). She also trained five times per week for 16 weeks for 40 minutes per session, with hand, lower limb, and body weight exercises (calf raises and half-squats), and gait training was performed (Figure 2). DPd therapy [10] (daratumumab (1,800 mg/body; days one and eight), pomalidomide (1 mg/body; days one to eight), and dexamethasone (4 mg/body; days one and eight)) was started on April 26, 2022, after the systemic skin rash, which was a side effect of the DLd therapy, disappeared. However, a systemic skin rash reappeared on May 3, 2022. Therefore, DPd therapy was discontinued on day nine. The first course of DBd therapy [11] (daratumumab (1,800 mg/body; days one, eight, and fifteen), bortezomib (1.6 mg/body; days one, eight, and eleven), dexamethasone (8 mg/body; days one, eight, and fifteen)) was started on June 7, 2022, after the systemic skin rash, a side effect of DPd therapy, disappeared (Figures 2-4). Bone marrow aspiration from the right posterior iliac crest was performed on June 27, 2022. Bone marrow aspirate smear showed that plasma cells accounted for 0.6% of nucleated cells (Figure 1, Panel B). The second course of DBd therapy (daratumumab (1,800 mg/body; days one and eight), bortezomib (1.6 mg/body; days one, four, eight, and eleven), dexamethasone (8 mg/body; days one, four, eight, and fifteen)) was started on June 28, 2022. Serum and urine protein electrophoreses were performed on July 18, 2022. Serum protein electrophoresis showed M protein, (Ig)G-κ M proteinemia, which was daratumumab (anti-CD38 monoclonal antibodies) itself, and no (Ig)D-λ M protein. Urine protein electrophoresis showed no Bence-Jones proteins (Figure 1, Panels Ci, Cii). A bone marrow aspiration from the right posterior iliac crest was performed on July 18, 2022, and the bone marrow aspirate smear showed that plasma cells accounted for 0.4% of nucleated cells (Figure 1, Panel Ciii). Karyotypic analysis of the bone marrow cells revealed 46, XY. Thus, the patient achieved a complete response (CR). During hospitalization, the serum Alb level remained >3.2 g/dL, and her body weight, BMI, ASM, hand grip strength, Cr, and eGFR remained almost unchanged. Her Hb level and six-meter walking speed slightly increased, while the serum HbA1c did not decrease to normal values (Figures 3, 4). The patient had no adverse effects other than a skin rash during treatment.

Case two

**Figure 5:**
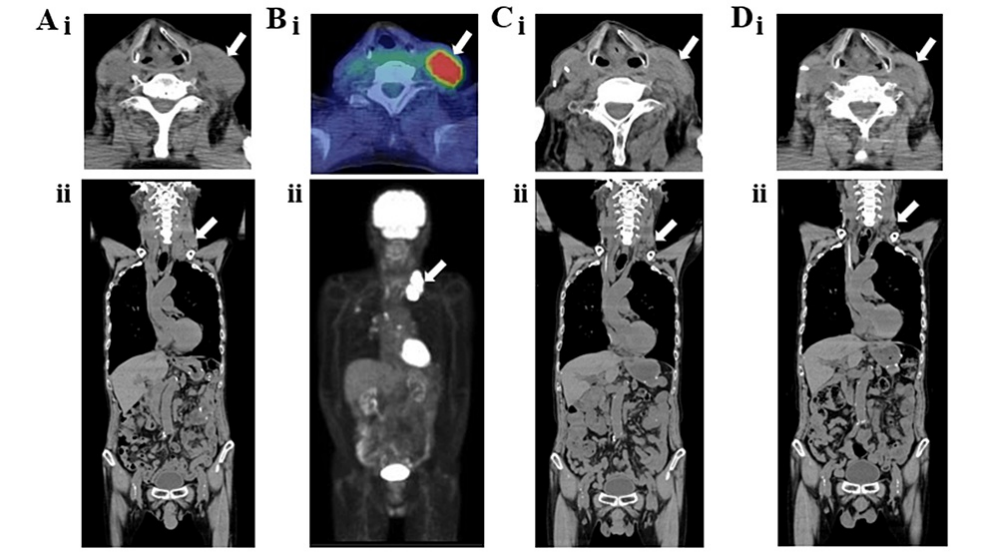
Radiological findings in Case two. (A) CT findings on June 23, 2022. (i) Fifth cervical vertebrae finding and (ii) systemic finding. (B) PET-CT findings on July 20, 2022. (i) Fifth cervical vertebrae finding and (ii) systemic finding. (C) CT findings on September 22, 2022. (i) Fifth cervical vertebrae finding and (ii) systemic finding. (D) CT findings on November 2, 2022. (i) Fifth cervical vertebrae finding and (ii) systemic finding. Arrows indicate left cervical lymphadenopathy CT: computed tomography; PET-CT: positron emission tomography-computed tomography

An 86-year-old man was referred to our hospital from the Department of Internal Medicine of another hospital on June 23, 2022, to investigate the cause of left cervical lymphadenopathy and elevated soluble interleukin-2 receptor (sIL-2R) levels. He had been diagnosed with classical Hodgkin lymphoma (lymphocyte-rich, stage IV in Ann Arbor Classification) at another hospital in 2011 and had undergone six courses of ABVD (doxorubicin/bleomycin/vinblastine/dacarbazine) therapy [12] to obtain and maintain CR. At presentation, the patient was afebrile. Physical examination revealed left cervical lymphadenopathy, no skin changes, and no hepatosplenomegaly. A complete blood test revealed anemia (Hb = 10.1 g/dL; normal range = 13.6-18.3 g/dL). Additionally, serum sIL-2R (1,635 U/mL; normal range = 122-496 U/mL) and Cr (1.25 mg/dL; normal range = 0.65-1.09 mg/dL) increased. However, eGFR (42.9 mL/minute/L; normal range ≥89.0 mL/minute/L]) decreased. Serum Alb (3.8 g/dL; normal range = 3.7-5.5 g/dL), CRP (0.07 mg/dL; normal range = 0.00-0.30 mg/dL), and LDH (1,961 U/mL; normal range = 120-245 U/mL) were normal. Computed tomography (CT) revealed a left cervical tumor (Figure 5, Panels Ai, Aii).

**Figure 6:**
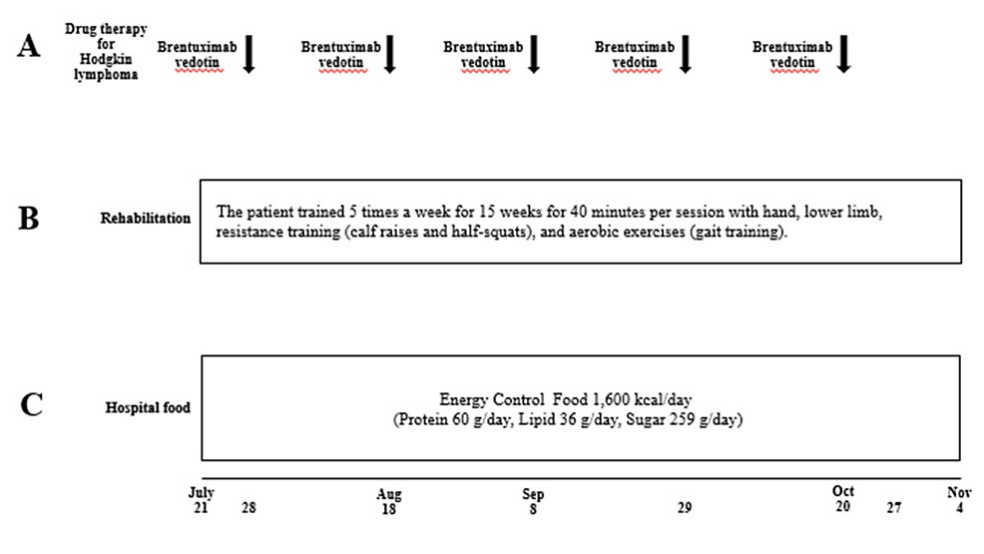
Drug therapy for Hodgkin lymphoma, rehabilitation, and nutritional therapy in Case two. (A) Drug therapy for Hodgkin lymphomas. (B) Rehabilitation protocol. (C) Nutritional therapy using hospital food.

The swollen left neck lymph node was biopsied, and the patient was histopathologically diagnosed with classical Hodgkin lymphoma (lymphocyte-rich). Upper gastrointestinal endoscopy and colonoscopy showed no lymphoma lesions. PET-CT revealed lymphoma cell infiltration into the left cervical lymph nodes (Figure 5, Panels Bi, Bii). However, bone marrow biopsy from the bilateral posterior iliac crest showed no lymphoma cell infiltration. The patient was diagnosed with stage IA disease according to the Lugano classification and was hospitalized on July 21, 2022 (Figure 6). His height, body weight, and ideal body weight were 157.0 cm, 46.0 kg, and 54.4 kg, respectively. We investigated the presence of sarcopenia using the AWGS 2019 criteria. The ASM, hand grip strength, and six-meter walking speed were 6.5 kg/m^2^, 27.2 kg, and 1.46 m/second, respectively. The AMS and hand grip strength were reduced, and the patient was diagnosed with sarcopenia. Next, we investigated the presence of malnutrition using the GLIM criteria. The malnutrition risk is screened using the MUST [3]. The patient’s MUST score was 3. His weight had remained unchanged over a one-year period, and his BMI was 18.7 kg/m^2^. Therefore, he was diagnosed with severe malnutrition.

As the patient was diagnosed with severe malnutrition and sarcopenia, a rehabilitation program combining exercise and nutritional intervention was initiated, and chemotherapy was implemented (Figure 6).

**Figure 7:**
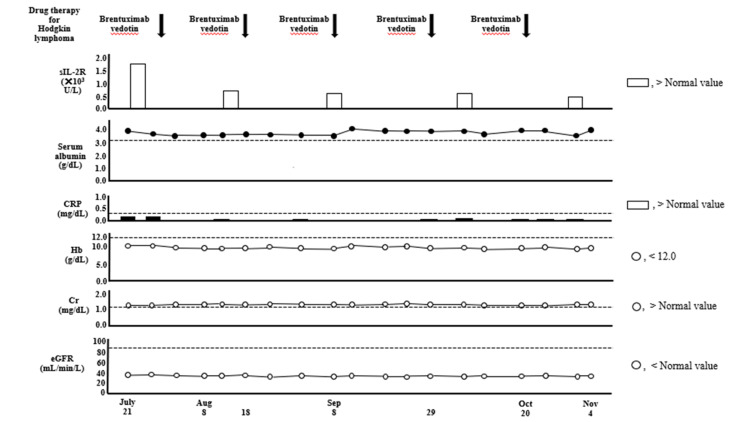
Data of sIL-2R, serum Alb, CRP, Hb, Cr, and eGFR in Case two. sIL-2R: soluble interleukin-2 receptor; Alb: albumin; Cr: creatinine; CRP: C-reactive protein; eGFR: estimated glomerular filtration rate; Hb: hemoglobin

**Figure 8:**
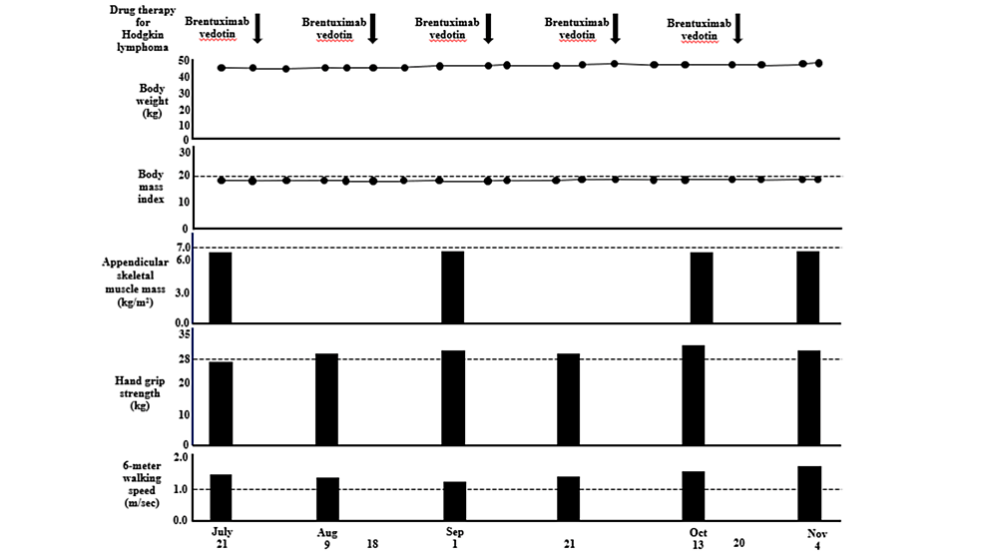
Data of body weight, body mass index, and the Asian Working Group for Sarcopenia 2019 consensus diagnostic criteria in Case two. The criteria for low appendicular skeletal muscle mass is <7.0 kg/m^2^ in men by bioelectrical impedance analysis; the criteria for low muscle strength is defined as hand grip strength <28 kg for men; the criteria for low physical performance is six-meter walking speed <1.0 m/second.

He was treated with intravenous brentuximab vedotin every three weeks [13] as salvage chemotherapy for relapsed classical Hodgkin lymphoma (Figures 6-8). Brentuximab vedotin was started at a 20% reduced dose and was gradually increased to the full dose of 1.80 mg/kg, as the patient had developed neutropenia and pneumonia from full-dose ABVD therapies. The brentuximab vedotin doses on July 28, August 18, September 8, September 29, and October 20, 2022, were 1.44 mg/kg, 1.62 mg/kg, 1.62 mg/kg, 1.80 mg/kg, and 1.80 mg/kg, respectively. We administered nutritional therapy (30 kcal/kg ideal body weight/day of energy, 1.1 g/kg ideal body weight/day of protein) using hospital food. The patient consumed energy control food (1,600 kcal/day and 60 g protein/day). He also trained five times per week for 15 weeks for 40 minutes per session, with hand, lower limb, and body weight exercises (calf raises and half-squats), and gait training was performed (Figure 6). As the number of treatments with brentuximab vedotin increased, the swollen left cervical lymph nodes shrank, and sIL-2R levels decreased (Figure 5, Panels Ci, Cii, Di, Dii, Figure 7).

During hospitalization, the patient regained hand grip strength, the serum Alb level remained >3.2 g/dL, body weight, BMI, ASM, six-meter walking speed, Cr, and eGFR remained almost unchanged, and Hb did not increase to normal values (Figures 6-8).

The cause of anemia was diagnosed as renal anemia. The patient had no adverse effects other than mild numbness of the fingers during the treatment.

## Discussion

Adverse outcomes associated with malignancy-related malnutrition have been widely reported, including, but not limited to, a decrease in treatment tolerance and increased mortality [4,14]. Additionally, sarcopenia in patients with malignancies has been associated with severe chemotherapeutic toxicity, changes in body composition (including reduced muscle mass secondary to onco-specific treatments), and worse clinical outcomes [5,6,15].

We attempted nutritional rehabilitation to eliminate malnutrition and sarcopenia. There are no established exercise and nutritional regimens for managing malnutrition, as defined by the GLIM criteria, or sarcopenia as defined by the AWGS 2019 criteria. Furthermore, there have been no reports on how malnutrition and sarcopenia can be improved in patients with multiple myeloma and Hodgkin lymphoma. Moreover, nutritional therapy with exercise prescriptions for men with prostate cancer is thought to offer a long-term, multi-health benefit for managing cancer-related fatigue [16]. Nutritional rehabilitation may increase muscle mass and strength in older patients with sarcopenia [17]. Energy requirements at rest are recommended to be 25-30 kcal/kg/day, in line with the oncology-specific European Society of Parenteral and Enteral Nutrition guidelines [18]; however, energy requirements during rehabilitation are not described in the guidelines. To maintain and regain lean body mass and function in older people (>65 years), the PROT-AGE study group recommends a protein intake of at least 1.0-1.2 g/kg/day [19]. Furthermore, most older adults with acute or chronic diseases require even more dietary protein (i.e., 1.2-1.5 g/kg/day). The European Society of Parenteral and Enteral Nutrition guidelines on protein intake in cancer do not address a low muscle mass, being given in a range of 1.0-1.5 g/kg/day [18]. Based on the above background, we speculated that a diet containing more than 30 kcal/kg/day of energy and more than 1.2 g/kg/day of protein is useful for nutrition therapy against malnutrition, as defined by GLIM criteria, and sarcopenia, as defined in the AWGS 2019 criteria. Furthermore, most older adults with acute or chronic diseases require even more dietary protein (i.e., 1.2-1.5 g/kg/day). Case one was administered 40 kcal/kg/day of energy and 1.5 g/kg/day of protein, and Case two was administered 35 kcal/kg/day of energy and 1.3 g/kg/day of protein. We believe that the BMIs of our patients were appropriately maintained through nutritional therapy. Our data indicated that energy requirements during rehabilitation might be 30 kcal/kg ideal body weight/day (35-40 kcal/kg/day) in hematological malignancy patients with malnutrition and sarcopenia. Our data also indicated that protein intake during rehabilitation might be 1.1 g/kg ideal body weight/day (1.3-1.5 g/kg/day) of protein in hematological malignancy patients with malnutrition and sarcopenia. Our patients trained five times a week for 15-16 weeks for 40 minutes per session, with hand, lower limb, and resistance training (calf raises and half-squats), as well as aerobic exercises (gait training). Consequently, the six-meter walking speed was slightly increased in Case one, and she could maintain her ASM and hand grip strength. In contrast, Case two recovered hand grip strength and was able to maintain his ASM and six-meter walking speed. Yoshimura et al. [20] showed that a program to carry out comprehensive training using resistance exercises twice a week for three months, for 60 minutes per session, might increase ASM and walking speed in older patients without malignancies. As our rehabilitation staff has Saturdays and Sundays off every week, rehabilitation was carried out for five days (five times) other than Saturdays and Sundays. We previously reported the usefulness of nutritional rehabilitation for sarcopenia in a patient with diffuse large B-cell lymphoma [7]. The patient regained hand grip strength and maintained ASM. However, the effects of nutritional rehabilitation on GLIM-defined malnutrition in hematological malignancies have not yet been reported. Our data in this case report showed that rehabilitation might be effective in ASM maintenance, hand grip strength recovery, and increasing the six-meter walking speed in hematological malignancy patients with malnutrition and sarcopenia.

Furthermore, because malnutrition, as defined by the GLIM criteria, and sarcopenia are considered adverse prognostic factors for older adult patients with hematological malignancies [4], hematologists should evaluate the existence and severity of malnutrition and sarcopenia in patients before starting appropriate therapy. Hematologists should also understand how to treat patients with hematological malignancies with malnutrition and sarcopenia. Our combination of chemotherapy and nutritional rehabilitation may overcome malnutrition and sarcopenia in patients with hematological malignancies.

Our case series has some limitations. We provide empirical data that two older hematological malignancy patients for whom nutrition rehabilitation was effective against GLIM-defined malnutrition and sarcopenia. Our case series was a retrospective analysis with a small sample size and no long-term survival data. In the future, we intend to conduct a prospective study to clarify these limitations.

## Conclusions

This is the first report showing that nutritional rehabilitation might be effective against GLIM-defined malnutrition and sarcopenia in patients with hematological malignancies. In our view, hematologists should evaluate the existence and severity of malnutrition and sarcopenia in patients before starting chemotherapy. Furthermore, we strongly hope that a clinical trial be conducted to investigate whether nutritional rehabilitation is effective against GLIM-defined malnutrition and sarcopenia in older hematological malignancy patients.
